# Unrevealing the Mystery of Latent Leishmaniasis: What Cells Can Host *Leishmania*?

**DOI:** 10.3390/pathogens12020246

**Published:** 2023-02-03

**Authors:** Andrea Valigurová, Iva Kolářová

**Affiliations:** 1 Department of Botany and Zoology, Faculty of Science, Masaryk University, Kotlářská 2, 611 37 Brno, Czech Republic; 2 Department of Parasitology, Faculty of Science, Charles University, Albertov 6, 128 44 Prague, Czech Republic

**Keywords:** *Leishmania*, latent leishmaniasis, host cell invasion, non-phagocytic host cell, fibroblast, adipocyte, mesenchymal stem cell, phagocytosis, membrane repair, lysosomes

## Abstract

*Leishmania* spp. (Kinetoplastida) are unicellular parasites causing leishmaniases, neglected tropical diseases of medical and veterinary importance. In the vertebrate host, *Leishmania* parasites multiply intracellularly in professional phagocytes, such as monocytes and macrophages. However, their close relative with intracellular development—*Trypanosoma cruzi*—can unlock even non-professional phagocytes. Since *Leishmania* and *T. cruzi* have similar organelle equipment, is it possible that *Leishmania* can invade and even proliferate in cells other than the professional phagocytes? Additionally, could these cells play a role in the long-term persistence of *Leishmania* in the host, even in cured individuals? In this review, we provide (i) an overview of non-canonical *Leishmania* host cells and (ii) an insight into the strategies that *Leishmania* may use to enter them. Many studies point to fibroblasts as already established host cells that are important in latent leishmaniasis and disease epidemiology, as they support *Leishmania* transformation into amastigotes and even their multiplication. To invade them, *Leishmania* causes damage to their plasma membrane and exploits the subsequent repair mechanism via lysosome-triggered endocytosis. Unrevealing the interactions between *Leishmania* and its non-canonical host cells may shed light on the persistence of these parasites in vertebrate hosts, a way to control latent leishmaniasis.

## 1. Background

*Leishmania* (Kinetoplastida: Trypanosomatidae) can be considered a highly successful unicellular parasite that specialises in the phagocytic cells of the mammalian immune system. Infection is initiated by vector-delivered flagellated promastigotes that are engulfed by the mammalian host cells within the skin tissue, where they transform and replicate as round intracellular amastigotes, typically within monocytes and macrophages. The infection is then maintained by replicating amastigotes capable of infecting other host cells. The established amastigote infection manifests as leishmaniasis, a spectrum of diseases with clinical outcomes dependent on multiple factors, including parasite species and virulence, host genetic background, and immune status [1].

Clinical symptoms can usually be detected once the parasite is established in the mammalian host cells; however, some of infected individuals remain asymptomatic with subclinical infection [2]. Moreover, *Leishmania* parasites can persist within host tissues even after treatment or self-healing [2,3,4]. In all these scenarios, the latent persistence of *Leishmania* parasites poses a threat to the patient, as they may reactivate during immunosuppression [2]. Therefore, the key questions are how do *Leishmania* parasites persist in mammalian tissues and where do they replicate?

According to textbook knowledge, *Leishmania* is an intracellular parasite that can only enter professional phagocytic cells. Lacking the tools, such as an apical complex, that help apicomplexan parasites (e.g., *Plasmodium* or *Toxoplasma*) to actively penetrate the host cell, *Leishmania* helps itself by binding opsonins to its surface, being nicely buttered to persuade the phagocyte for phagocytosis [5]. The secret of the *Leishmania* parasites survival success may lie in the range of suitable host cells, since monocytes and macrophages are not the only mammalian cells that can host these parasites. In addition to these canonical host cells, there is increasing evidence that *Leishmania* amastigotes can reside in other cell types [6,7], including fibroblasts and epithelial cells (e.g., [7,8,9,10,11,12,13,14,15]). The invasion of cells other than professional phagocytes can affect the dynamics of *Leishmania* infection in two different ways. First, it can provide the parasite with a safe temporary shelter for the parasite to evade host immunity before reaching monocytes/macrophages as the primary host cells. Second, these cells may also serve as a reservoir for leishmaniasis recrudescence [8,9]. The presence of latent *Leishmania* stages in cells such as fibroblasts, adipocytes, or adipose-tissue mesenchymal stem cells may explain the ability of this parasite to survive for decades after self-healing or treatment. The risk of *Leishmania* reactivation due to immunosuppression should be kept in mind, especially for patients in endemic areas undergoing transplantation or co-infected with HIV [2,16].

In this review, we provide (i) an overview of non-canonical *Leishmania* host cells and (ii) an insight into the strategies used by *Leishmania* parasites to invade the host cell.

## 2. Professional Phagocytes as the *Leishmania* Primary Host Cells

Professional phagocytes of the mammalian immune system (e.g., neutrophils, macrophages, monocytes, dendritic cells, eosinophils, and mast cells) play an essential role in homeostasis and inflammation, enabling pathogen clearance and host tissue healing [17,18]. They are equipped with phagocytic receptors (Toll-like receptors, complement receptors, Fc-gamma receptors, scavenger receptors, etc.) and compounds involved in intracellular killing. The phagocyted material is enclosed in a vesicle called a phagosome, which is intended to fuse with lysosomes for the hydrolytic degradation of the phagosome contents. If the phagosome contains a pathogenic cargo, it can also be eliminated via oxidative burst and the production of reactive nitrogen species, including nitric oxide (NO) as a potent leishmanicidal molecule [1].

Depending on the phase of *Leishmania* infection, different professional phagocytes are involved in *Leishmania* survival and host defence. Immediately after transmission, *Leishmania* parasites have been found in tissue-resident macrophages and, within a few hours post infection, also in neutrophils [6]. These two cell types are the first host cells for *Leishmania*; however, the promastigotes appear to parasitise them without apparent multiplication [6]. Tissue-resident macrophages and neutrophils thus rather provide a temporary shelter for promastigotes during the first hours of infection [6,19,20].

In contrast, tissue-resident mast cells are also capable of engulfing *Leishmania* promastigotes, and even supporting their transformation and multiplication [21]. However, the exact role of mast cells in leishmaniasis depends on the infecting *Leishmania* species and the genetic background of the host [22,23].

Later on, within a few days, *Leishmania* parasites infect other myeloid cells such as inflammatory monocytes, monocyte-derived dendritic cells, and also eosinophils [6]. Eosinophils and dendritic cells participate in the local inflammatory response, which also shapes the onset of adaptive anti-*Leishmania* immunity [23,24]. In addition, the increased migration of infected dendritic cells may contribute to parasite dissemination and the visceralisation of leishmaniasis [25].

During the subsequent chronic phase of infection, neutrophils infected with amastigotes are able to support *Leishmania* multiplication. It appears that neutrophils do not support *Leishmania* transformation from promastigote to amastigote, but once infected with the amastigote form, *Leishmania* is able to multiply there, as has been shown for *L. amazonensis* [26,27].

Multiple cell invasion mechanisms have been attributed to *Leishmania* to invade professional phagocytes. Passive entry into macrophages appears to be based on the ligand-receptor mediated interactions between the promastigote and macrophage surface molecules [5]. Contrary to textbook knowledge, *Leishmania* promastigotes are also able to enter the host cell by an active process using the host cell plasma membrane repair mechanism [8,28,29]. The details of the passive and active entry of *Leishmania* into the professional phagocytes are discussed in the following two subchapters.

### 2.1. Passive Entry of Leishmania into the Host Cell

Passive host cell entry is a widely accepted mode of *Leishmania* internalisation. *Leishmania* was previously thought to be completely dependent on the phagocytic activity of its host cell, facilitating the phagocytosis by the deliberate binding of host cell opsonins. Passive entry of *Leishmania* into the professional phagocytes, such as macrophages and monocytes, is generally considered to be a receptor-mediated phagocytosis involving molecules such as *Leishmania* ligands, host cell receptors, and optionally also host-derived opsonins (reviewed in detail, e.g., in [5,30,31]).

**Figure 1:**
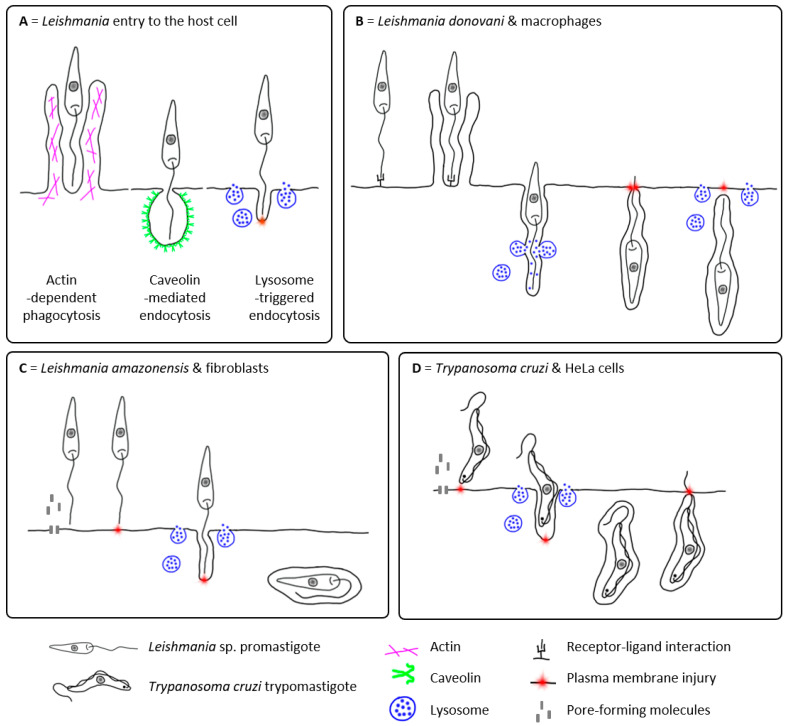
Interactions of *Leishmania* sp. and *Trypanosoma cruzi* with the host cell. Three possible entry pathways of *Leishmania* promastigotes into the host cell as discussed in this review (**A**) and models of *Leishmania* sp. and *Trypanosoma cruzi* interactions with different host cells involving lysosome-triggered endocytosis (**B**–**D**). (**A**) The most widely accepted models of *Leishmania* promastigote entry into the host cell are actin-dependent phagocytosis (magenta) and caveolin-mediated endocytosis (green). In the newly proposed model of lysosome-triggered endocytosis (blue), *Leishmania* cause injury (red) to the host cell plasma membrane and exploit the subsequent repair mechanism based on the lysosome exocytosis, which facilitates the endocytosis of the damaged plasma membrane together with the *Leishmania* promastigotes. (**B**) *Leishmania donovani* metacyclic promastigotes preferentially enter primary bone marrow-derived murine macrophages via the flagellar tip, presumably in a receptor-ligand mediated pathway. During the internalisation, lysosomes fuse with the forming phagosome. Prior to complete engulfment, the motile promastigote inside the incomplete parasitophorous vacuole reorients the flagellar tip towards the macrophage plasma membrane, in some cases even protruding out of the host cell. During this phase, the flagellar motility causes damage to the plasma membrane leading to lysosome exocytosis, followed by increased endocytosis. Complete internalisation is accompanied by the loss of promastigote motility and the phagolysosome is located close to the host cell nucleus. Lysosome exocytosis during the later phase of promastigote internalisation appears to promote host cell survival rather than the host cell invasion process itself (as proposed in [29]). (**C**) *Leishmania amazonensis* metacyclic promastigotes enter murine fibroblasts (mouse embryonic cell line) via a non-phagocytic pathway dependent on lysosome exocytosis. Prior to internalisation, promastigotes induce host cell membrane injury by an unknown mechanism, probably involving flagellar motility and/or *Leishmania*-derived pore-forming cytolysins. Membrane damage and the associated increase of intracellular Ca^2+^ lead to lysosome exocytosis, followed by increased endocytosis. After internalisation, *Leishmania* is enclosed in ceramide-rich endocytic vacuoles. In contrast to macrophages, the lysosome exocytosis facilitates the host cell invasion process itself (as proposed in [8]). (**D**) *Trypanosoma cruzi* trypomastigotes enter epithelial HeLa cells via a non-phagocytic pathway dependent on lysosome exocytosis. Prior to internalisation, trypomastigotes cause injury to the host cell membrane by an unknown mechanism, probably involving parasite motility and/or *Trypanosoma*-derived pore-forming toxins. Membrane damage and the associated increase in intracellular Ca^2+^ lead to lysosome exocytosis. Acid shingomyelinase released from lysosomes hydrolyses sphingomyelin on the outer membrane leaflet to ceramide, leading to increased ceramide-driven endocytosis of the injured plasma membrane together with trypomastigotes. After internalisation, *T. cruzi* is found in ceramide-rich endocytic vacuoles. In some cases, engulfed trypomastigotes move towards the host cell plasma membrane, protruding their flagella out of the host cell. During this event, the flagellar motility causes additional damage to the plasma membrane, leading to increased endocytosis. Lysosome exocytosis facilitates the host cell invasion process itself and supports host cell survival by restoring membrane integrity (as proposed in [38]).

However, phagocytosis is only one of the several processes by which the cell takes up extracellular material. In general, the process of endocytosis can be divided into four basic categories: (i) actin-mediated endocytosis (including phagocytosis and micropinocytosis), (ii) caveolin-dependent endocytosis, (iii) clathrin-dependent endocytosis, and (iv) clathrin/caveolin-independent endocytic pathways [32]. Several studies have shown that *Leishmania* is more likely to be internalised by caveolin-dependent endocytosis (Figure 1A) [33,34]. It is mediated by caveolins, integral membrane proteins that preferentially oligomerise in cholesterol-rich lipid rafts to form the membrane invagination. Through the caveolin-dependent endocytosis, the uptake of extracellular material can also be mediated by specific receptors [32,33]. Caveolin-mediated internalisation of *Leishmania* promastigotes has been observed in murine macrophages for *L. chagasi* and *L. donovani* [33,34,35,36,37]. Furthermore, caveolin-coated phagosomes showed delayed fusion with lysosomes, allowing the promastigote to transform into the amastigote form, which is better adapted to survive in acidified phagolysosomes [35]. However, this delay has only been observed in virulent metacyclic promastigotes [36,37]. Engulfment of avirulent or serum-opsonised promastigotes [36,37] or amastigotes [33,35] appears to be caveolin-independent, showing no delay in phagosome fusion with lysosomes [35]. Although this lack of the delay is fatal for promastigotes, amastigotes are, on the other hand, already adapted to the phagolysosomal microenvironment and can survive and proliferate there [33].

Clathrin-dependent endocytosis does not appear to play a role in the uptake of *Leishmania* promastigotes [34].

### 2.2. Active Entry of Leishmania into the Host Cell?

Surprisingly, *Leishmania* may also be able to enter host cells actively. The flagellar motility of promastigotes enables them to actively participate in phagocytic uptake by the macrophage (Figure 1B) [29]. This is most likely made possible by the polarised phagocytosis of *Leishmania* promastigotes induced by the interaction between their flagellar tip and the invaded cell, leading to the formation of pseudopodia. Pseudopodia begin at the tip of the parasite’s flagellum and extend towards its cell body [29]. The incessant activity of the promastigote flagellum leads to the reorientation of the parasite within the macrophage, with the flagellum pointing towards the host cell periphery. The oscillations of *Leishmania* parasites may even cause local damage to the host cell plasma membrane, leading to the Ca^2+^-dependent recruitment of host lysosomes to the site of the parasite invasion and their subsequent exocytosis, which is involved in the host cell plasma membrane repair process, as also observed in *Trypanosoma cruzi* invading HeLa cells (Figure 1D) [5,29,38].

The exocytosis of lysosomes during the *T. cruzi* internalisation occurs by their fusion with the host plasma membrane adjacent to the parasite, inducing the release of acid sphingomyelinase (ASM) and the production of ceramide by the hydrolysis of membrane sphingomyelin. The production of ceramide in the outer leaflet of the membrane induces endocytosis of the injured membrane, which the parasite uses for its own internalisation into the newly formed lysosomal endosomes [5,38]. In contrast to *T. cruzi*, *Leishmania*-mediated lysosome exocytosis has been reported to occur after the parasite uptake by the macrophage during its intracellular oscillating movement (Figure 1B) [29]. Since its close relative *T. cruzi* uses lysosome exocytosis for the invasion process itself [5,38,39], could *Leishmania* use a similar strategy to invade cells other than professional phagocytes?

## 3. Other Potential Host Cells of *Leishmania*

There is increasing evidence that *Leishmania* can invade or even survive and multiply in other cells that are not considered to be professional phagocytes. To enter these cells, *Leishmania* can use both passive as well as active strategies (Figure 1A).

Although the phagocytic process is more commonly associated with professional phagocytes, it has been shown that almost all cells in the human body are capable of phagocytosis (including skin fibroblasts) and caveolin-dependent endocytosis (including adipocytes, endothelial, and muscle cells) [18,40,41]. Entry into host cells by a process similar to classical phagocytosis has been documented, for example, in Chinese hamster ovary cell lines co-incubated with *L. amazonensis* amastigotes [42].

However, *Leishmania* may also be actively involved in the entry process into the non-canonical host cells, including but not limited to fibroblasts, adipocytes, myofibres or epidermal cells, such as pigmented cells (e.g., [8,43,44,45,46]). Since it is well documented that its close relative *T. cruzi* uses lysosome exocytosis to invade the host cell, including the non-professional phagocytic cells [5,38,39], it is likely that *Leishmania* could use a similar strategy [8,28].

While in macrophages, *Leishmania*-mediated lysosome exocytosis has only been reported after the parasite uptake (Figure 1B) [29]; in different host cells, it can facilitate the invasion process itself (Figure 1C) [8,28]. This appears to be the case at least for *L. amazonensis* entry into the fibroblasts [8]. The colocalisation of *L. amazonensis* promastigotes that have half-entered a fibroblast with the LAMP marker suggests that lysosome recruitment does indeed occur concomitantly with parasite invasion and that lysosomes donate their membrane to form the nascent parasitophorous vacuole [8]. Two hypotheses have been proposed for the *L. amazonensis* initiation of the host cell plasma membrane damage leading to lysosome exocytosis and the subsequent endocytosis in these host cells [8]: (i) parasite flagellar motility towards the host cell plasma membrane, causing mechanical damage (as in *T. cruzi* [38]) and/or (ii) the secretion of cytolytic molecules (such as pore-forming cytolysins) leading to plasma membrane permeabilisation [47,48].

Other possible strategies that *Leishmania* parasites might use to invade non-professional phagocytes should be verified in the future. It is even possible that the promastigote flagellum itself plays a much more important role in invasion than previously thought. According to older in vitro observations, the flagellum could serve as an anchor inside the invaded cell, by which the parasite pulls itself inside when penetrating cells with limited phagocytic capacity [43]. In fact, *Leishmania* parasites appear to possess several mechanisms that would allow them to enter their host cells, and it is likely that these mechanisms may alternate or even complement each other depending on the type of the cell attacked and the infecting *Leishmania* species.

It is obvious that *Leishmania* parasites have tools to invade even the non-canonical host cells that have been neglected as players in the dynamics of *Leishmania* infection and its persistence. These cells are listed in Table 1 and include, besides other professional phagocytes (mast cells, dendritic cells, eosinophils, and histiocyte-like cell lines), mainly non-professional phagocytic cells, such as lymphocytes, fibroblasts, adipocytes, epithelial and endothelial cells, mesenchymal stem cells, myocytes, and keratinocytes.

To collect the data for Table 1, we adapted the tables of Rittig and Bogdan (2000) [7] and Chang and Fish (2017) [49], which we further expanded and updated using the following strategy. A literature search of relevant articles was conducted between December 2022 and January 2023 using databases such as Web of Science and PubMed. The search was performed independently by both authors using combinations of keywords, such as this Boolean string: “Leishmania* AND (fibroblast* OR adipo* OR fat OR “epithelial cell*” OR epithel* OR myocyte* OR muscle* OR myofibre OR “stem cell*” OR keratinocyte* OR “endothelial cell*” OR endothel*) AND (multiplicat* OR transform* OR uptake OR internali* OR phagocyto* OR intracellular* OR amastigote*)”. The search was not restricted by the year of publication. The retrieved articles were selected based on the eligibility criteria including, but not limited to: an original research article, full text access, detailed description of methodology, host cells of vertebrate origin, and clear statement of results. Exceptionally, secondary citations were used for research articles with a unique output, but not accessible in the full text version. References in selected articles were also evaluated. The *Leishmania* species names listed in Table 1 correspond to the names provided in the original research articles, as older papers, in particular, did not use more accurate molecular techniques for species identification, and it is therefore not possible to complement them with current taxonomy and nomenclature.

The data listed in Table 1 indicate that the outcome of *Leishmania* internalisation is likely to depend on the host and *Leishmania* species, the infecting form of *Leishmania* (promastigote vs. amastigote), and also the origin of the host cell or tissue. This table only includes vertebrate host cells; however, some *Leishmania* species have also been found to invade insect cell lines [50,51,52]. Of particular interest is that the cell lines prepared from mosquitoes (*Aedes aegypti*) or sand flies (*Lutzomyia spinicrassa*) and mainly containing cell types with epithelial and fibroblast appearance, also support the internalisation of *Leishmania* promastigotes, together with their transformation into the amastigote form [50,51] and even their multiplication [52].

**Table 1:** Non-canonical host cells of *Leishmania* parasites.

Host Cell/Origin	*Leishmania* Species		Main Outcome	Detection Method	Reference
**PROFESSIONAL PHAGOCYTES**					
**Dendritic cells (DCs)**					
Langerhans cells from mouse skin ^❖^	*L. major* PMs		No or low PMs uptake	LM/Diff-Quik, TEM, FL/AO+EtBr	[53,54,55]
	*L. major* AMs		AMs uptake and internalisation, no or weak multiplication	LM/Diff-Quik, TEM, FL/AO+EtBr, ICC	[54,56,57]
Mouse lymph node DCs ^♦○^	*L. major* AMs		Presence of AMs	LM/G, IHC	[56,58,59]
Mouse spleen DCs ^❖^	*L. major* PMs/AMs, *L. m. mexicana* PMs		PMs/AMs uptake	LM/G	[59,60]
Mouse bone marrow DCs ^❖^	*L. major* PMs/AMs, *L. mexicana* PMs, *L. amazonensis* AMs/PMs		PMs/AMs uptake in all; multiplication reported only in *L. amazonensis*	LM/Diff-Quik, ICC, FC	[61,62,63,64]
	*L. infantum* PMs/PMs (CFSE)		PMs uptake, transformation into AMs	LM/G, FC/CFSE-PMs	[65]
Human immature monocyte-derived DCs^❖^	*L. amazonensis*, *L. braziliensis*, *L. infantum* PMs		PMs uptake, internalisation	CLSM/Dapi	[25]
	*L. donovani* PMs		PMs uptake, transformation into AMs	LM/MGG	[66]
**Mast cells (MCs)**					
Mouse peritoneal MCs ^❖^	*L. tropica*, *L. donovani* PMs (CFSE)		PMs uptake in *L. tropica*, but not in *L. donovani*	FC+CLSM/CFSE-PMs	[22,67]
Mouse bone marrow MCs ^❖^	*L. major*, *L. infantum* PMs		PMs uptake, transformation into AMs, multiplication leading to cell lysis and AMs release	LM/MGG	[21]
**Eosinophils**					
Human peripheral eosinophils ^❖^	*L. donovani* PMs		PMs uptake and killing after 2 h p.i.	LM/Diff-Quik	[68]
	*L. donovani* AMs		AMs uptake, not efficient killing	LM, TEM	[69]
Rat peritoneal eosinophils ^❖^	*L. major* PMs		PMs uptake and killing	LM/MGG, ICC	[70]
Rat peritoneal eosinophils ^♦^^○^^❖^	*L. m. amazonensis* PMs/AMs		PMs/AMs uptake and killing	TEM	[71]
Mouse eosinophils in skin lesion ^♦○^	*L. m. mexicana* AMs		AMs uptake, not efficient killing	TEM	[72]
**Histiocyte-like cells**					[7]
Sticker dog sarcoma 503 cells ^❖^	*L. donovani*, *L. mexicana, L. m. mexicana, L. braziliensis, L. b. pifanoi*, *L. t. major* PMs/AMs		PMs/AMs uptake, multiplication, continuous passages	LM/G, TEM	[73,74,75,76,77,78]
	*L. m. mexicana* PMs		PMs uptake, transformation into AMs, multiplication after day 3 p.i., transformation into PMs	LM/G, TEM	[43]
	*L. adleri*, *L. hoogstraali*, *L. agamae* PMs		Low PMs uptake, transformation into AMs	LM/G	[43]
**NON-PROFESSIONAL PHAGOCYTES**					
**Lymphocytes**					
Human B (Daudi) and T (HUT78) cells ^❖^	*L. donovani* PMs/AMs		PMs/AMs uptake, PMs transformation into AMs, viability up to 2 weeks after infection with AMs	LM/G, TEM	[79]
**Fibrocytes**					
Mouse peripheral blood fibrocytes ^❖^	*L. amazonensis* PMs		PMs uptake, transformation into AMs, low multiplication, clearance by 72 h p.i.	LM/G, FL/Dapi, TEM, SEM	[80]
**Fibroblasts**					
Canine skin fibroblasts ^♦□^	*Leishmania* sp.		Presence of AMs	TEM, LM/HE, G, PAS	[81]
	*L. donovani*		Presence of AMs	IHC	[82]
Human skin fibroblasts ^♦□^	*L. tropica*		Presence of AMs	LM/G, TEM	[83,84]
	*Leishmania* sp. (cutaneous)		Presence of AMs	TEM	[85]
Human skin fibroblasts ^❖^	*L. amazonensis* PMs		PMs uptake, transformation into AMs, multiplication	TEM	[14]
	*L. m. amazonensis* AMs		AMs uptake, multiplication, killing of AMs by day 8 p.i.	LM/G, TEM, ICC	[12]
	*Leishmania* sp. (mucocutaneous), *L. donovani* PMs		PMs uptake in *Leishmania* sp. (not in *L. donovani*), transformation into AMs, no or low multiplication, decline during a 3-week period p.i.	LM, TEM, SEM	[86]
Human foreskin fibroblasts ^❖^	*L. donovani* PMs		PMs uptake, transformation into AMs, no multiplication, viability up to day 14 p.i.	LM, TEM, SEM	[87]
	*L. major* PMs (SPIONs)		PMs uptake	LM/SPIONs-PMs+Prussian blue, TEM	[88]
	*L. major* PMs (AO, Dil)		PMs uptake	FL/AO-PMs, Dil-PMs	[89]
Mouse skin fibroblasts ^♦○^	*L. amazonensis* PMs		AMs presence	LM/HE, Lennert’s G	[90]
Mouse skin fibroblasts ^❖^	*L. major* PMs/AMs		PMs/AMs uptake	ICC	[9]
	*L. amazonensis* PMs		PMs uptake and killing of PMs after day 3 p.i.	LM/G, TEM, FL/Dapi	[91]
	*L. infantum*, *L. mexicana* PMs		PMs uptake, transformation into AMs, multiplication	LM/G, TEM	[10]
Hamster skin fibroblasts ^❖^	*L. infantum*, *L. mexicana* PMs		PMs uptake, transformation into AMs, multiplication	LM/G, TEM	[10]
Rat skin fibroblasts ^❖^	*L. infantum*, *L. mexicana* PMs		PMs uptake, transformation into AMs, no multiplication	LM/G, TEM	[10]
Human fibroblasts in lymph node ^♦□^	*Leishmania* sp.		AMs presence	LM/G	[92]
Mouse fibroblasts in lymph node ^❖^	*L. major* PMs/AMs		PMs/AMs uptake	TEM, ICC	[9]
Draining lymph nodes of healed mice (presumably fibroblasts) ^♦○^	*L. major* PMs		Presence of AMs, parasite survival or limited killing	IHC	[9]
Mouse embryonic fibroblasts ^❖^	*L. donovani* PMs		PMs uptake, transformation into AMs, efficient host defence via IFN-inducible guanylate binding proteins	LM/G, CLSM/Dapi	[93]
	*L. amazonensis* PMs (RFP)		PMs uptake, transformation into AMs, multiplication	LM/HE, TEM, CLSM+FC/RFP-PMs	[8]
	*L. major* PMs		PMs uptake	CLSM/Dapi	[94]
Mouse tumour fibroblasts (L cells) ^❖^	*L. amazonensis*, *L. major* AMs (GFP)		AMs uptake, internalisation (low in *L. major*), multiplication (not in *L. major*)	CLSM/GFP-AMs	[15]
Fibroblasts from embryonic chick brain ^❖^	*L. donovani* AMs		AMs uptake, viability up to day 17 p.i., transformation into PMs	LM/G	[11]
Fibroblast-like cells from embryonic chick muscle ^❖^	*L. donovani*/presumably AMs		AMs uptake, no multiplication, degeneration after day 20 p.i.	LM/HE	[95]
Mouse perineurial cells ^♦○^	*L. amazonensis* PMs		Presence of AMs	TEM	[96]
**Adipocytes**					
Mouse brown and white adipose tissue ^♦○^	*L. infantum* PMs		PMs uptake, viable AMs present for up to 40 weeks p.i.	IHC, qPCR	[46]
Mouse adipocytes derived from primary pre-adipocytes from subcutaneous white adipose tissue ^❖^	*L. infantum* PMs (GFP)		PMs uptake (further progress not reported)	TEM, qPCR, CLSM/GFP-AMs	[46]
Human adipocytes derived from adipose tissue primary progenitor cells ^❖^	*L. infantum* PMs (GFP)		PMs uptake (further progress not reported)	qPCR, CLSM/GFP-AMs	[46]
Mouse adipocytes differentiated in vitro from 3T3-L1 fibroblasts ^❖^	*L. amazonensis, L. braziliensis* PMs/AMs		PMs/AMs uptake, PMs transformation into AMs, viability up to 144 h p.i. and ability to transform into PMs	LM/G, FL/Dapi, TEM	[45]
	*L. amazonensis* AMs (GFP)		AMs uptake, viability up to 144 h p.i.	FL/GFP-AMs	[45]
**Epithelial cells**					
Human epithelial cells of eccrine sweat gland (HIV patient) ^♦□^	*Leishmania* sp., *L. infantum*		AMs presence	LM/HE	[97,98]
Human retinal pigmented epithelial cells (ARPE-19) ^❖^	*L. amazonensis* PMs		PMs uptake, internalisation	LM/G, IHC, TEM	[99]
Human amnion epithelium ^❖^	*L. donovani*, *L. b. pifanoi* PMs		PMs uptake, transformation into AMs, clearance by day 29–32 p.i.	LM/G	[100]
	*L. donovan* PMs		PMs uptake, transformation into AMs, multiplication (not clear whether PMS or AMs)	LM/G	[101]
A549 (human adenocarcinomic alveolar basal epithelium) cells ^❖^	*L. donovani* PMs		PMs uptake, transformation into AMs, efficient defence via IFN-inducible guanylate binding proteins	LM/G, CLSM/Dapi	[93]
HeLa (human cervix carcinoma) cells ^❖^	*L. t. major* PMs		PMs uptake, transformation into AMs, multiplication, destruction of host cells after day 3	LM	[102]
	*L. donovani* PMs		PMs uptake, transformation into AMs, decline after 5 h p.i.	LM/G	[103]
LLC-MK2 (rhesus monkey kidney epithelium) cells ^❖^	*L. donovani* AMs		AMs uptake, multiplication	LM/G	[104]
Vero (monkey kidney) cells ^❖^	*L. chagasi*, *L. braziliensis* PMs		PMs uptake, transformation into AMs, multiplication	LM/G, TEM	[105,106]
Chinese hamster ovary cells ^❖^	*L. amazonensis* AMs		AMs uptake, multiplication	IHC, TEM	[42]
*C. burnetii*-infected Vero cells ^❖^	*L. amazonensis* AMs		AMs uptake, multiplication	LM, TEM	[107]
*C. burnetii*-infected Chinese hamster ovary cells ^❖^	*L. amazonensis* AMs		AMs uptake, multiplication	LM, TEM, CLSM/PI	[107,108]
**Mesenchymal stem cells (MSCs)**					
Mouse bone marrow MSCs ^♦○❖^	*L. infantum* PMs		PMs uptake, transformation into AMs	LM/G, ICC, FC	[109]
Human adipose tissue MSCs ^❖^	*L. donovani*, *L. infantum*, *L. major*, *L. tropica* PMs		PMs uptake, transformation into AMs but AMs present only at day 1 p.i.; at day 7, 14, 21, and 28 only PMs detected	LM/G, microcapillary culture method, PCR	[16]
**Myocytes**					
Canine skeletal/smooth muscles ^♦□^	*L. infantum*, *Leishmania* sp.		Presence of AMs within myofibres	LM/HE, IHC	[110,111]
Mouse skeletal muscles ^♦○^	*L. amazonensis* AMs		Presence of AMs within myofibres	LM/HE	[112]
Turtle heart cells ^❖^	*L. m. mexicana*, *L. adleri*, *L. hoogstraali* PMs		PMs uptake (lower in *L. adleri* and *L. hoogstraali*), transformation into AMs (further progress not reported)	TEM (*L. mexicana* only), LM/G	[43]
**Endothelial cells**					
Human endothelial cells of blood vessels ^♦□^	*L. donovani*, *Leishmania* sp.		Presence of AMs	LM	[81,113,114]
Human endothelial cells of capillaries ^♦○^	*L. tropica* PMs		Presence of AMs	LM	[115]
Human microvascular endothelial (HMEC-1) cell line ^❖^	*L. infantum* PMs		No uptake of PMs	LM/G	[116]
**Keratinocytes**					
Human keratinocytes (HIV patient) ^♦□^	*L. infantum*		AMs presence	LM/HE	[98]
Human keratinocytes ^❖^	*L. infantum, L. major* PMs		PMs uptake, transformation into AMs at low levels, no multiplication	LM/G, CLSM/Dapi	[117]
**Unidentified cells in primary cultures**					
Hamster kidney cells ^❖^	*L. braziliensis*, *L. donovani* PMs		PMs uptake, transformation into AMs, multiplication (not in *L. donovani*)	LM/G	[118,119], as cited in [7,49]
Chicken embryo muscles ^♦○^	*L. t. major* PMs		PMs uptake, transformation into AMs, multiplication, destruction of host cells after day 3	LM	[102]

Abbreviations: AMs—amastigotes, AO—acridine orange, CFSE—carboxyfluorescein N-succinimidyl ester, CLSM—confocal laser scanning microscopy, DCs—dendritic cells, EtBr—ethidium bromide, FC—flow cytometry, FL—fluorescence microscopy, G—Giemsa, GFP—green fluorescent protein, HE—haematoxylin-eosin, ICC—immunocytochemistry, IHC—immunohistochemistry, LM—light microscopy, MCs—mast cells, MGG—May–Grünwald–Giemsa, MSCs—mesenchymal stems cells, PAS—periodic acid-Schiff, PCR—polymerase chain reaction, PI—propidium iodide, PMs—promastigotes, p.i.—post inoculation, qPCR—quantitative polymerase chain reaction, RFP—red fluorescent protein, SEM—scanning electron microscopy, SPIONs—superparamagnetic iron oxide nanoparticles, TEM—transmission electron microscopy. Symbols: ^❖^—in vitro, ^♦^—in vivo, ^□^—clinical case, ^○^—experimental infection. Note: *Leishmania* species names correspond to the names as provided in the original research articles.

In the following subchapters, we have focused on studies using host cells that are as close as possible to their natural state, since cancerous or mutated cells (such as those used for immortalised cell lines) may have altered metabolism that may also affect Leishmania entry, survival, and multiplication.

### 3.1. Fibroblasts

Fibroblasts may play a neglected role in latent leishmaniasis and the disease epidemiology because, in some host-parasite combinations, they can support intracellular parasite survival, transformation into amastigotes, and even amastigote multiplication, resulting in viable progeny capable of transforming back to promastigotes [8].

Several in vitro and in vivo studies have reported that fibroblasts harbour *Leishmania* amastigotes (Table 1) [8,9,10,11,14,81,83,84,85,86,87]. Conflicting results have been observed regarding the survival of *L. amazonensis* in fibroblasts, ranging from limited survival of parasites (e.g., [12,91]) to their successful multiplication [8,10,14,15] (Table 1). Co-incubation of enucleated fibroblasts (cytoplasts, cell nucleus artificially removed in vitro) with *L. amazonensis* revealed that the parasitophorous vacuole biogenesis and parasite multiplication are independent of the host cell nucleus, and showed these cells to be a promising model for studies focusing on the role of the host cell nucleus during the parasite-host interactions (in particular, the modulation of the gene expression) [15]. The amastigotes of *L. amazonensis* have also been detected in the perineurial cells (=epithelioid myofibroblasts) of BALB/c mice with experimental cutaneous leishmaniasis [96]. It is interesting that *L. mexicana* and *L. infantum* were able to multiply in mouse and hamster skin fibroblasts, but not in skin fibroblasts from rats [10]. In contrast, *L. donovani* does not appear to be capable of long-term survival and multiplication in fibroblasts in any of the host species tested—human [87], mouse [93], nor chicken [11,95] (Table 1).

Ultrastructural and immunohistochemical analysis of skin biopsies from dogs with naturally acquired leishmaniasis revealed the presence of free and occasionally vacuole-enclosed amastigotes within fibroblasts, while some amastigotes in close contact with the host plasma membrane appear to damage it, indicating that amastigotes also have an active invasion potential [81]. The authors speculated that the source of these amastigotes infecting fibroblasts in the deeper layers of the skin were necrotic macrophages. Similarly, *L. donovani* amastigotes have been seen surrounded by a closely applied membrane of human foreskin fibroblasts cultured in vitro [87].

The potential involvement of fibroblasts in the pathogenesis of cutaneous leishmaniasis is of particular interest, as recent studies indicate their role in *Leishmania* immune evasion strategies [13]. Fibroblasts are one of the most abundant cells at the site of transmission and are important producers of macrophage- and neutrophil-attracting chemokines. Fibroblasts also interact directly with macrophages during wound healing and can move by diapedesis, thus being capable of *Leishmania* dissemination [8]. The relatively long lifespan of fibroblasts and their limited ability to eliminate invaders could lead to the persistence of infection [7]. In addition, fibroblasts are capable of phagocytosis but have a limited ability to control the *Leishmania* infection through NO production [9]. Indeed, in healed mice, approximately 40% of *L. major* amastigotes were found in skin and draining lymph node fibroblasts, indicating that they may serve as a safe shelter and a site of potential recrudescence [9,58]. In latent leishmaniasis, the balance may be maintained by neighbouring macrophages producing enough NO to destroy *Leishmania* amastigotes within the fibroblasts [9].

Similar to professional phagocytes, the entry of *Leishmania* into fibroblasts could be either passive or active (Figure 1A). An older in vitro study, supported by transmission electron microscopic visualisation, claimed that the infection of fibroblasts by *L. braziliensis* promastigotes occurs via the parasite-induced phagocytosis, when the parasites enter fibroblasts with their flagellar end through pseudopodia-like formations on the host cell surface [86]. The engulfed promastigotes settled in vacuoles that did not fuse with secondary lysosomes and transformed into amastigotes. Uptake is likely to be receptor-mediated, as skin fibroblasts cocultured with *L. amazonensis* promastigotes showed an increased expression of the mannose receptor during the early stages of infection, possibly binding mannosylated ligands on the promastigote surface [91]. This modulation of fibroblast mannose receptor expression was reversed concomitantly with the loss of parasite viability, indicating that the presence of viable parasites is required to maintain it [91].

However, the active entry of *Leishmania* into the fibroblasts has also been reported (Figure 1). It has been shown that *L. amazonensis* induces its entry into host fibroblasts by damaging the fibroblast plasma membrane, leading to the internalisation of promastigotes during the subsequent membrane repair process (Figure 1C) [8]. The invasion process is independent of host actin remodelling (i.e., this process is not a form of induced phagocytosis) and involves Ca^2+^-dependent recruitment/exocytosis of host lysosomes to repair the plasma membrane. During this process *Leishmania* actively induces lysosome-triggered endocytosis, a cell invasion mechanism based on the transient permeabilisation of the host cell plasma membrane [8]. Similar to *T. cruzi* interactions with HeLa cells [38], this invasion process has only been observed with the viable metacyclic stage of the parasite; neither dead metacyclic nor the procyclic promastigotes (the developmental stage of *Leishmania* found only in the vector) were engulfed by fibroblasts [8]. On the other hand, parasite-unrelated membrane injury promotes internalisation of *L. amazonensis* promastigotes [8] as well as *T. cruzi* trypomastigotes [38].

### 3.2. Adipocytes

Adipose tissue has also been postulated as a potential reservoir for intracellular pathogens with the ability to induce disease relapses [46]. Indeed, recent studies have confirmed the ability of *Leishmania* promastigotes to infect adipocytes in vitro and in vivo [45,46]. Adipose tissue could serve as a perfect reservoir for *Leishmania*, especially considering that this tissue niche is also used by its relatives—extracellular *T. brucei* [120] and intracellular *T. cruzi* [121]. Although their survival strategies are not entirely the same as those of *Leishmania*, we may still find some parallels that can inspire future research. *Trypanosoma brucei* has been shown to accumulate in the adipose tissue of mice early after infection [120]. These adipose tissue extracellular *T. brucei* forms, which are transcriptionally distinct from bloodstream forms, can replicate and are capable of infecting a naïve host [120]. Moreover, trypomastigotes of *T. cruzi* invade human and mouse adipocytes and transform into amastigotes during the acute phase of infection [121]. Although replication in adipocytes has not yet been directly observed in *Leishmania*, *L. amazonensis*, and *L. brasiliensis* amastigotes recovered from infected cells retain the ability to differentiate into replicative promastigotes [45], and recovered *L. infantum* amastigotes were infectious to another host [46].

### 3.3. Mesenchymal Stems Cells

Mesenchymal stems cells (MSCs) residing in bone marrow could also provide a perfect protective niche for *Leishmania* parasites and support their persistence in the host organism. Among other factors, these cells are (i) capable of self-renewal and have low-reactive oxygen species properties (ideal for long-term parasite viability), (ii) do not normally express MHC Class II on their surface and their MHC Class I molecules do not trigger effector functions of cytotoxic T-cells, and (iii) express potent drug efflux pumps (probably enabling *Leishmania* drug evasion) [109]. Indeed, *L. infantum* promastigotes successfully infected the CD271+/Sca1+ bone marrow MSCs of C57BL/6 mice and transformed into amastigotes in both in vivo and in vitro settings [109]. Moreover, several *Leishmania* species, including agents of both the cutaneous and visceral leishmaniases (Table 1), have been shown to persist for some time in an inactive form in cultures of adipose tissue-derived MSCs [16]. As stem cells generally remain dormant in the absence of an exogenous stimulus, they may represent ideal reservoir host cells for *Leishmania* [16]. The mechanism of invasion of mesenchymal stem cells remains to be elucidated, although phagocytic properties have already been reported for adipose tissue MSCs [16].

### 3.4. Myocytes

Muscle cells are highly parasitised by *T. cruzi* [122] but are overlooked in *Leishmania* studies. It is therefore of particular interest that *L. infantum* amastigotes have been detected in the muscle biopsies from dogs with obvious muscle damage, causing a progressive polymyositis affecting the masticatory and skeletal muscles [110]. Moreover, another study reported canine leishmaniasis associated with myositis of adnexal, extraocular and intraocular smooth and striated muscles that were parasitised by *Leishmania* amastigotes [111].

In addition, there are in vitro studies reporting the internalisation and replication of several *Leishmania* species in muscle cells of different origins [43,102]. The ability of *Leishmania* amastigotes to invade muscle fibres was also confirmed by an in vivo experimental study comparing the muscle infection by *L. amazonensis* in two mouse strains with a different susceptibility to leishmaniasis—susceptible BALB/c mice and resistant C3H.He mice [112]. While the BALB/c mice showed an intense inflammatory infiltrate between the amastigote-infected myofibres, followed by a total muscle destruction at day 90 p.i., the C3H.He mice showed only a mild inflammatory infiltrate without intracellular amastigotes, followed by a muscle repair process [112].

**Figure 2:**
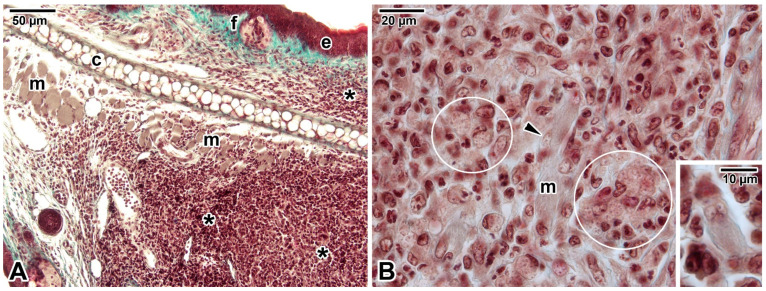
Putative involvement of myocytes in cutaneous leishmaniasis shown in sectioned ear pinna of a female BALB/c mouse infected with *Leishmania major*. Histological sections stained with green Masson’s trichrome. (**A**) *Leishmania* lesion in the ear pinna. (**B**) Detailed view showing infected macrophages (some of which are encircled) and muscle cells with putative amastigotes (arrowhead and the inset). *Asterisk*—lesion with massive cell infiltration, *arrowhead*—putative amastigotes, *c*—cartilage, *e—*epidermis, *f*—hair follicle, *m*—muscle, *white circles*—infected macrophages. The involvement of myocytes in cutaneous leishmaniosis requires further confirmation using more specific detection methods.

In BALB/c mice experimentally inoculated with *L. major* promastigotes, we also occasionally observed the presence of amastigote-like structures within muscle fibres (Figure 2, unpublished results). However, these observations require confirmation by transmission electron microscopy or immunolabelling.

The mechanism of *Leishmania* entry into muscle cells remains to be elucidated. While older studies hypothesised the ability of promastigotes to actively penetrate target cells through the motility of their flagellum acting as an anchor [43], others speculate that *Leishmania* parasites could penetrate myocytes, rich on fucose-mannose ligands, using the fucose-mannose receptor [112]. Parasite-unrelated injury to muscle cells may facilitate *Leishmania* uptake via lysosome-triggered endocytosis during repair of the damaged plasma membrane, as has been shown for *L. amazonensis* and fibroblasts [8].

### 3.5. Endothelial Cells

Endothelial cell parasitism by *Leishmania* remains unclear, as only a few studies (some of them older) with conflicting results are currently available [113,115,116,123,124]. However, similar to *T. cruzi* [122], it is likely that at least some *Leishmania* species could be able to infect endothelial cells.

While a recent in vitro analysis of a human microvascular endothelial cell line (HMEC-1) co-incubated with *L. infantum* promastigotes reported the absence of internalised parasites [116], the intracellular localisation of *Leishmania* has been reported from endothelial cells lining the blood-vessels of the kidney, liver, and colon in visceral leishmaniasis [81,113,114,123]. However, it should be noted that the accurate localisation of amastigotes in routine histopathology (usually used as the sole detection method in older studies) is challenging and may be less sensitive than immunolabelling, as demonstrated in canine cutaneous leishmaniasis [125]. For example, the histopathological examination of a human subcutaneous nodule after experimental inoculation with *L. tropica* promastigotes revealed the abundant presence of amastigotes within the endothelial cells lining the capillaries near the centre of the lesion [115], but another study reported that *L. braziliensis* parasites were more likely to be attached to the wall of dermal blood vessels and free in the capillary lumen [124]. Accordingly, the L-SIGN receptor, specifically expressed in liver sinusoidal endothelial cells, acts as a receptor for viscerotropic *L. infantum* (but not dermotropic *L. pifanoi*), resulting in the strong binding of amastigotes to the cultured endothelial cells; however, no invasion of these cells was reported [126].

### 3.6. Keratinocytes

As one of the most abundant epidermal cell types and an important source of immunomodulatory signals in the skin, keratinocytes may play a key role in the early stages of insect-borne diseases, including leishmaniasis. These cells appear to be unsuitable for *Leishmania* replication, the function of keratinocytes being more immunomodulatory. Although human keratinocytes were shown to internalise *L. infantum* or *L. major* promastigotes in vitro at low levels, they did not allow efficient amastigote multiplication [117]. Accordingly, several in vivo or ex vivo studies reported the absence of keratinocytes parasitised by *Leishmania* [55,96,127]. Nevertheless, keratinocytes exposed to extracellular *L. infantum* and *L. major* parasites have been shown to alter their transcriptional signatures and appear to be stimulated to release factors that influence monocyte infection [117]. The authors hypothesised that the pro-inflammatory response of keratinocytes induced by *L. infantum* may limit the local survival of the parasite in the skin environment and thus promote its dissemination, whereas the ‘silent’ interaction of *L. major* with keratinocytes may increase its ability to survive locally, leading to cutaneous leishmaniasis [117]. It has therefore been proposed that keratinocytes may initiate or suppress the pro-inflammatory response at the site of infection, thereby influencing tissue pathology [117].

## 4. Conclusions and Perspectives

*Leishmania* well-recognised primary host cells are professional phagocytes, macrophages, and monocytes, but other cell types might also be infected with *Leishmania* parasites. The presence of *L. major* transcripts has been shown to be associated with multiple cell types at the site of infection. In addition to the well-known host cells of the myeloid lineage (macrophages, inflammatory monocytes, neutrophils, and dendritic cells), they are also found in endothelial and epithelial cells, fibroblasts, keratinocytes, chondrocytes, and myoblasts [128]. However, the key requirement for the host cell—to support *Leishmania* survival and multiplication—has only been proven for a few of them (e.g., [8,10,46]).

The most studied non-canonical *Leishmania* host cells are fibroblasts [8,9,10,11,12,13,14,15,81,82,83,84,85,86,87,88,89,90,91,92,93,94,95,96]. They can migrate within the skin tissue [8], potentially allowing *Leishmania* to spread from the site of transmission, thereby disseminating the infection and enhancing the possibility of being engulfed by the vector during blood feeding. Due to their relatively long lifespan and low leishmanicidal activity, fibroblasts may serve as an ideal reservoir host cell in latent cutaneous leishmaniasis [9]. In visceral leishmaniasis, adipocytes may also play this role [45,46]. The putative reservoir host cells for latent infection should ideally support the intracellular survival of *Leishmania* for a prolonged period, ideally also facilitating amastigote replication. If the cell cannot support *Leishmania* replication, the amastigote may leave the reservoir host cell, multiply in monocytes or macrophages, and find safe shelter in another reservoir host cell. Such a scenario may be possible since *Leishmania* parasites are not dormant during latent leishmaniasis but are continuously replicating [129], being under the tight immune control of macrophage-derived NO [58].

The promiscuity of *Leishmania* with respect to host cells has been demonstrated using the cell lines of invertebrate origin [50,51,52], showing the ability of *Leishmania* parasites to also infect—under artificial conditions—the cells of the insect vector [51], where they naturally occur only extracellularly [130,131]. In humans, such artificial conditions could be induced, for example, by HIV immunosuppression, leading to an unusual and rare localisation of *Leishmania* within sweat gland epithelial cells in the host dermis [97,98]. To our knowledge, such localisation has not been observed in immunocompetent individuals (Table 1). Moreover, the hidden promiscuity of *Leishmania* parasites may have unexpected therapeutic implications, affecting the screening of new drug candidates. The widely used in vitro testing on promastigotes (vector-derived developmental stage) [132] may not reveal the full complexity of amastigote presence in different tissues/cells during the mammalian host infection. Pharmacokinetics and pharmacodynamics would be better evaluated in the context of multiple *Leishmania* host cells.

The ability of *Leishmania* to invade different cell types may also affect the epidemiology of leishmaniasis, being another factor to be consider during the transmission from the mammalian host to the insect vector. *Leishmania* is able to persist in the uninflamed skin of the mammalian host, while preserving its infectious potential for sand fly vectors [133]. As these parasites can gradually accumulate in the skin, even in clinically healthy hosts, and remain infectious to their insect vectors [133], it is necessary, at least in endemic areas, to monitor not only cured patients but also potential reservoirs, such as dogs or asymptomatic humans.

*Leishmania* internalisation into the host cell is well described in professional phagocytes, but the mechanisms in non-canonical host cells are less well understood. The mode of *Leishmania* entry into the host cells, of whatever type, appears to be multifactorial, depending on the host cell type, *Leishmania* developmental stage (promastigotes vs. amastigotes), *Leishmania* virulence, as well as on the immune status of the host (e.g., the presence of anti-*Leishmania* antibodies as opsonising agents). The *Leishmania* internalisation could be receptor-mediated (e.g., [5,30,31,91]), where *Leishmania* appears to be passively engulfed, or actively initiated by *Leishmania* promastigote by wounding the host cell plasma membrane [8], e.g., via the movement of its flagellar tip [29,43]. At least three possible entry pathways have been described (Figure 1): (i) actin-dependent phagocytosis, (ii) caveolin-mediated endocytosis, and (iii) lysosome-triggered endocytosis associated with the host cell plasma–membrane repair mechanism [7,8,28,32,33,34,36,37].

Undoubtedly, more studies are needed to reveal all the details of the interactions between *Leishmania* and its various host cells in the hope of finding a way to better control leishmaniasis, including its latent form.

## Data Availability

No new data were created or analysed in this study. Data sharing is not applicable to this article.

## Abbreviations

AMs	Amastigotes
AO	Acridine orange
ASM	Acid sphingomyelinase
CFSE	Carboxyfluorescein N-succinimidyl ester
CLSM	Confocal laser scanning microscopy
DCs	Dendritic cells
EtBr	Ethidium bromide
FC	Flow cytometry
FL	Fluorescence microscopy
G	Giemsa
GFP	Green fluorescent protein
HE	Haematoxylin-eosin
HMEC-1	Human microvascular endothelial cell line
ICC	Immunocytochemistry
IHC	Immunohistochemistry
LAMP	Lysosomal membrane-associated protein
LM	Light microscopy
L-SIGN	Liver/lymph node-specific ICAM-3 grabbing nonintegrin
MCs	Mast cells
MGG	May–Grünwald–Giemsa
MSCs	Mesenchymal stems cells
NO	Nitric oxide
PAS	Periodic acid-Schiff
PCR	Polymerase chain reaction
PI	Propidium iodide
PMs	Promastigotes
p.i.	Post inoculation
qPCR	Quantitative polymerase chain reaction
RFP	Red fluorescent protein
SEM	Scanning electron microscopy
SPIONs	Superparamagnetic iron oxide nanoparticles
TEM	Transmission electron microscopy
